# Geographic risk modeling of childhood cancer relative to county-level crops, hazardous air pollutants and population density characteristics in Texas

**DOI:** 10.1186/1476-069X-7-45

**Published:** 2008-09-25

**Authors:** James A Thompson, Susan E Carozza, Li Zhu

**Affiliations:** 1 Department of Large Animal Clinical Science, Texas A&M University, College Station, Texas, 77843-4475, USA; 2 Department of Epidemiology and Biostatistics, School of Rural Public Health, Texas A&M University, College Station, Texas, 77843, USA

## Abstract

**Background:**

Childhood cancer has been linked to a variety of environmental factors, including agricultural activities, industrial pollutants and population mixing, but etiologic studies have often been inconclusive or inconsistent when considering specific cancer types. More specific exposure assessments are needed. It would be helpful to optimize future studies to incorporate knowledge of high-risk locations or geographic risk patterns. The objective of this study was to evaluate potential geographic risk patterns in Texas accounting for the possibility that multiple cancers may have similar geographic risks patterns.

**Methods:**

A spatio-temporal risk modeling approach was used, whereby 19 childhood cancer types were modeled as potentially correlated within county-years. The standard morbidity ratios were modeled as functions of intensive crop production, intensive release of hazardous air pollutants, population density, and rapid population growth.

**Results:**

There was supportive evidence for elevated risks for germ cell tumors and "other" gliomas in areas of intense cropping and for hepatic tumors in areas of intense release of hazardous air pollutants. The risk for Hodgkin lymphoma appeared to be reduced in areas of rapidly growing population. Elevated spatial risks included four cancer histotypes, "other" leukemias, Central Nervous System (CNS) embryonal tumors, CNS other gliomas and hepatic tumors with greater than 95% likelihood of elevated risks in at least one county.

**Conclusion:**

The Bayesian implementation of the Multivariate Conditional Autoregressive model provided a flexible approach to the spatial modeling of multiple childhood cancer histotypes. The current study identified geographic factors supporting more focused studies of germ cell tumors and "other" gliomas in areas of intense cropping, hepatic cancer near Hazardous Air Pollutant (HAP) release facilities and specific locations with increased risks for CNS embryonal tumors and for "other" leukemias. Further study should be performed to evaluate potentially lower risk for Hodgkin lymphoma and malignant bone tumors in counties with rapidly growing population.

## Background

Childhood cancer has been linked to a variety of environmental factors, including agricultural activities, industrial pollutants and population mixing, but etiologic studies have often been inconclusive or inconsistent when considering specific cancer types. More specific exposure assessments are needed. It would be helpful to optimize future studies to incorporate knowledge of high-risk locations or geographic risk patterns. Bayesian methods have begun to predominate disease mapping applications[1]. This emergence has been largely attributed to advances in computer hardware that have enabled Markov Chain Monte Carlo implementations of relatively complex Bayesian models[2] and recently developed software has made these techniques readily available to health researchers[3]. One of the potential advantages for performing the risk estimation in a Bayesian approach is that the inference is based on parameter or risk certainty and the risk can apply to the lower organizational unit, such as individuals, in a hierarchal Bayes approach [1]. Thus, the risk estimate would apply to an individual considering alternative living locations.

Pesticide exposure has long been implicated as a cause of childhood cancer and has been the focus of multiple studies, however, an unambiguous mechanistic cause-and-effect relationship has not been demonstrated [4]. Some studies whose objectives were to evaluate pesticide exposure used cropping intensity as an exposure surrogate and implicated farm or rural living as a positive risk factor [5]. These and other geographic studies have concentrated on geopolitical boundaries or buffers around point sources and have led to inconsistent results when each individual cancer type is considered among studies [6-10]. Even if an association was consistent, rural communities are different from urban communities in a great many ways, including population density characteristics and the extent of industrial pollution. Further research should be focused on high-risk areas to evaluate specific exposures and specific cancer types.

Hazardous air pollutants (HAP) have been linked to increased cancer risks for individuals living in close proximity to major point source HAP-releases. For example, childhood cancers and leukemias in Great Britain exhibited geographical clustering of birth places close to environmental hazards that included large scale combustion processes, processes using volatile organic compounds and waste incineration [11-13]. When areal source HAP were modeled at the census tract level, modeled values were related to leukemia rates in California [14]. Automobile exhaust is an area-source HAP that has received considerable scrutiny as a potential cause of childhood cancer. The studies have shown conflicting results and a critical review concluded that the weight of the epidemiological evidence indicates no increased risk for childhood cancer associated with exposure to traffic-related residential air pollution [15]. If surrogate exposure, like proximity to releases, is related to a rare disease, like childhood cancer, then investigation should focus on the higher risk locations.

Infectious causes of childhood cancer have been proposed and population characteristics of stability or mixing have been proposed and evaluated [16]. An Ohio study examined the geographic distribution of childhood leukemias relative to population density, population growth, and rural/urban locale. The study found higher rates for acute lymphocytic leukemia among the counties with most rapid population growth and the most urbanized counties had reduced risk for acute myeloid leukemia. The authors reasoned that the findings supported population mixing as a cause of some childhood cancers [17]. Mixing at the population level must have risks that can be estimated and communicated at the individual level. The risks for an individual to move or to be exposed to movers should be parsed and estimated in a more focused study.

The three types of proposed causal factors (cropping, HAP release and population density characteristics) are especially likely to be confounded in Texas where the spatial relationships between agricultural activity, industrial locations and characteristics of the population are especially complex. The objective of this study was to perform Bayesian geographical risk modeling of childhood cancer accounting for potential correlations among histotypes. Geographic patterns were assessed relative to county-level cropping intensity, intensive industrial releases of HAP and population density and growth. The goal of the study was to estimate the risk to an individual child based on specific characteristics of the mother's living location at the time of childbirth. Once higher risk locations are identified and characterized, more specific personal risk models can be developed.

## Methods

### Childhood cancer database

All Texas birth records from January 1, 1990 to December 31, 2002 were retrieved from the Texas Department of State Health Services (TDSHS). All births were followed for cancer incidence as reported to the Texas Cancer Registry (TCR) as of January 1, 2003. Therefore, a birth occurring January 1, 1990 had 13 years of follow-up and a birth on January 1, 2002 had one year of follow-up. The TCR is an active member of the North American Association of Central Cancer Registries (NAACCR) and follows the quality control guidelines and standards established by NAACCR (details available at the NAACCR website: ). The TCR estimates that cancer incidence data for the state are approximately 95% complete. Cancer diagnoses were grouped into 19 groups based on the most recent International Classification of Childhood Cancers (ICCC-3) [18]. Some pooling of very rare cancer types was performed as follows: childhood cancer subgroups Ic, Id and Ie were pooled and assigned the name "other leukemias"; subgroups IIb, IIc, IId and IIe were pooled into a single group and were labeled "non-Hodgkin lymphoma"; and subtypes IIIe, and IIIf were pooled into a group called "other CNS tumors." The database provided records for 3718 cancer cases distributed among 19 histotype groups and 3,805,745 total births.

### County-level agronomy practices

To evaluate annual crop production, data were retrieved from the Texas Almanac Characterization Tool Version 2.0.4 (Blackland Research and Extension Center, Texas Agricultural Experiment Station, Texas A&M University System, 720 East Blackland Road, Temple, TX, USA). By acreage, there are four major crops in Texas: corn, soybeans, wheat, and sorghum. When the combined total acres planted in these crops exceeded 20% of the county's total area, the county-year was classified as extensive cropping. The definition was chosen to identify the highest production locations but also to maintain an adequate number of high production county-years for estimation stability.

### County-level HAP

Hazardous air pollutants are substances that are known to be carcinogenic or to cause other serious health problems. The Environmental Protection Agency (EPA) currently identifies and records the release of 188 HAP. The data regarding Texas industries with air emissions of chemicals were available from the Toxic Release Inventory (TRI) program, a publicly available database of toxic chemical releases. This inventory was established under the Emergency Planning and Community Right-to-Know Act of 1986 (EPCRA) and expanded by the Pollution Prevention Act of 1990. The EPA compiles the TRI data each year and makes it available through several data access tools, including the TRI Explorer and Envirofacts. The data are available as either county emission summaries (county-level) or facility-specific emissions (point-source). Releases from four industries, petroleum refineries (Standard Industrial Code (SIC) Major Group 29), petroleum refining and related industries (SIC Major Group 33), chemical industries (SIC Major Group 28) and plastics production (SIC Major Group 30), were retrieved. The total releases were summed to identify high-release county-years. For year-to-year consistency, the list of 1988 core chemicals was used. A county-year in which 100 tonnes of toxic substances were released was considered to be high intensity HAP release. This definition identified the highest release county-years while maintaining enough intensive-release county-years for estimation stability.

### County-level population density

Counties were classified on population estimates from the US census bureau; the same source was used for estimates for intercensus years. County-years with populations of more than one million were classified as metropolitan and county-years with more than 50,000 residents were classified as urban. These are the standard definitions used by the U.S. census. County-years that showed population growth of more than one percent from the previous year were classified as rapid growth. The definition was chosen to be comparable to a recent study that evaluated a similar growth rate [17].

### Disease Modeling

The hierarchical modeling approach followed a general framework. The observed counts *Y*_*kij *_of childhood cancer histotype *k *in county *i *and year *j *were assumed to follow independent Poisson distributions conditional on an unknown mean *E*_*kij *_*exp(u*_*kij*_)

*Y*_*kij *_| *u*_*kij *_~ *Poisson(E*_*kij *_*exp(u*_*kij*_))

The expected count for histotype *k *in county *i*, and year *j *(*E*_*kij*_) was obtained by internal standardization from the given dataset such that the sum of observed cases for each histotype was exactly equal to the sum of expected cases for each histotype accounting for race. Race was defined as the mother's race as identified as one of four classes on the birth record: white, black, Hispanic and other. Year was defined as the calendar year of birth, 1990 to 2002, inclusive. Hence *exp*(*u*_*kij*_) is the standardized morbidity ratio (SMR). County-years with *exp*(*u*_*kij*_) > 1 had greater number of observed cancer cases than expected, and vice versa for counties with *exp*(*u*_*kij*_) < 1. The log-SMR *u*_*kij *_was modeled linearly for *k *= 1,..., 19 histotypes and *i *= 1,..., 254 counties and *j *= 1,...,13 years, as

*u*_*kij *_= *α*_*k *_+ *S*_*ki *_+ Year_*kj *_+ *β*1_k_*HAPS_*ij *_+ *β*2_k_*CROPS_*ij *_+ *β*3_k_*METRO_*ij *_+*β*4_k_*URBAN_*ij *_+ *β*5_k_*GROWTH_*ij*_

The *α*_*k *_represent the histotype-specific intercept terms for the baseline log-SMR across all counties and were assigned 19 independent flat priors. The *S*_*ki *_represent the county and histotype-specific log-SMR due to unmeasured or random county effects. The 19 × 254 dimensional matrix ***S ***was assigned a Multivariate Intrinsic Conditionally Autoregressive (MCAR) prior distribution with covariance matrix prior an inverse Wishart (***h***, ***R***) distribution with degrees of freedom ***h ***= 19 and ***R***, a 19 × 19 identity matrix. Year represented the risk for year of birth which contained the risk for the varying periods of observation and was assigned 19 independent random walk priors. Indicator variables (HAPS_ij_, CROPS_ij_, METRO_ij_, URBAN_ij _and GROWTH_ij_) were derived from the data as previously described for high intensity HAP release, high crop production, metropolitan, urban, and rapid population growth county-years, respectively. The *β*'s represented the log-relative risk for the county characteristics and were assigned a non-informative Normal prior distribution.

### Disease Mapping

The risk modeling was extended to derive overall spatial estimates for the 254 Texas counties from the 3302 county-years in the model previously described. Some of the geographic risk factors changed within a county from year to year. To evaluate each county's overall risk the mean expectation for each risk factor was calculated from the 13 years and used to estimate the county's overall risk attributable to the measured factors. The spatial model also adjusted risks for spatial associations and histotype correlations for the potential MCAR relationships that were estimated fully conditional upon all factors in the Disease Model, described previously. The parameterization used for spatial modeling was the posterior probability that the SMR estimate was greater than one [19]. This parameter is affected by both the magnitude and the precision of the SMR and was chosen to facilitate the objective of focusing further research on high-risk location and histotype combinations. The approach of establishing the probability of an increased risk is generally considered the first step for investigating a possible cluster and served the objective of identifying the locations with highest likelihood of elevated risk for further geographically focused studies. Spatial estimates were plotted using commercially available GIS software (ArcView^® ^GIS 3.2, Environmental Systems Research Institute, Inc., Redlands, CA).

### All modeling

All models employed Bayesian inference, with vague or flexible prior beliefs and an MCMC implementation. The MCMC implementation was performed by use of WinBUGS version 1.43 [3] and GeoBUGS version 1.2 [20]. The initial 1,000 iterations were discarded to allow for convergence and every hundredth of the following 100,000 iterations were sampled for the posterior distribution. The Bayesian estimate was taken as the posterior median of the parameter and 95% credible set was obtained from the posterior distribution quantiles. Observing convergence of two chains with widely different initial values for the random-effects precision parameters checked convergence to the posterior distribution.

## Results

**Figure 1:**
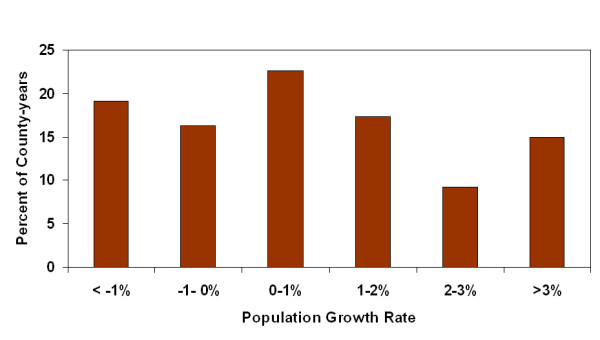
Frequency distribution of county-year population growth rates.

**Figure 2:**
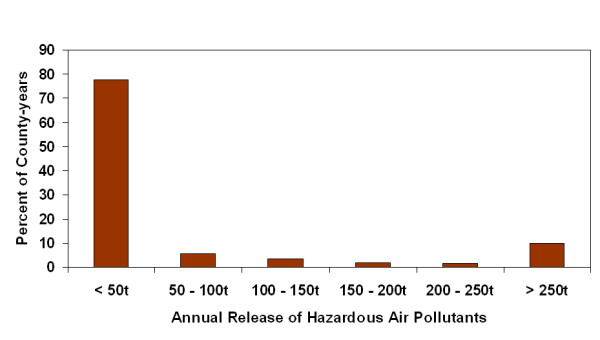
Frequency distribution of county-year release of hazardous air pollutants.

**Figure 3:**
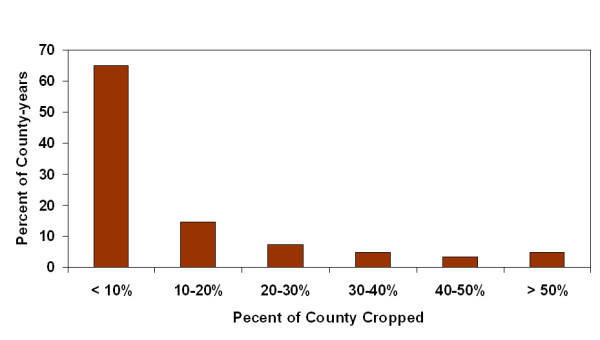
Frequency distribution of county-year cropping intensity for total corn, sorghum, wheat and cotton.

Two hundred and fifty four counties were modeled for 13 years providing 3302 county-years. The majority of county-years (79.1%) were classified as rural with a population of less than 50,000. For each year of the study there were exactly 4 metropolitan counties having more than one million residents: Bexar, Dallas, Harris and Tarrant counties. Population growth varied widely with population losses of more than 1% to population growth of greater than 4% both common. Growth of greater than 1% occurred in 41.7% of the county-years (Figure 1). The amount of HAP-release was commonly less than 50 tonnes per county-year but some very high releases were recorded, with 15.8% of the county-years having greater than 100 tonnes of release (Figure 2). Most county-years had less than 10% of the county area planted in corn, sorghum, cotton and wheat; however some county-years had greater than 50%, with 20.1% of the county-years having greater than 20% of the county cropped with these four crops (Figure 3).

**Figure 4:**
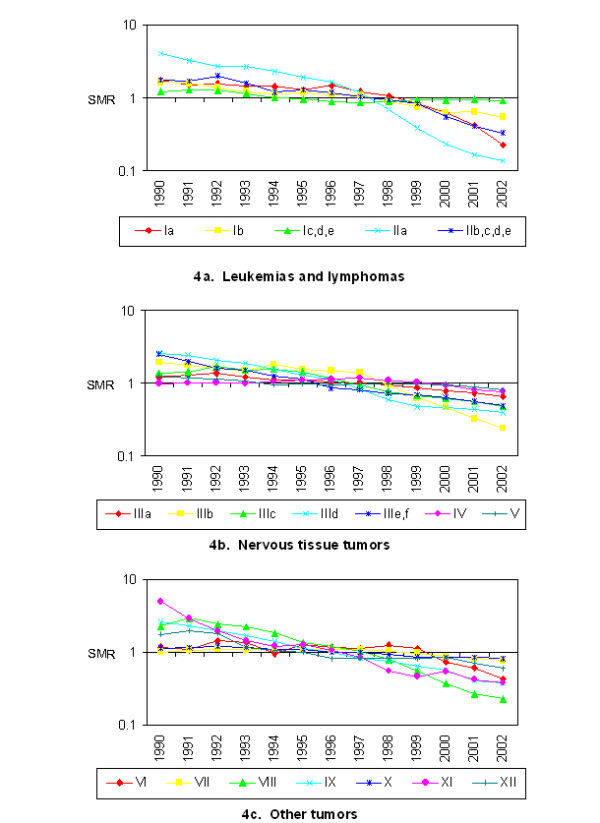
**Study-specific temporal effects**. 4a. Leukemias and lymphomas. 4b. Nervous tissue tumors. 4c. Other tumors. International Classification of Childhood Cancer (ICCC3) Classification Key. Ia. Acute Lymphoid leukemias (ALL). Ib. Acute myeloid leukemias (AML). Ic, d, e, Other leukemias. IIa. Hodgkin lymphoma. IIb, c, d, e. Non-Hodgkin lymphoma. IIIa. Ependymoma and choroid plexus tumor. IIIb. Astrocytomas. IIIc. Intracranial and intraspinal embryonal tumors. IIId. Other gliomas. IIIe, f Other CNS tumors. IV. Neuroblastoma and other peripheral nervous cell tumors. V Retinoblastoma. VI. Renal tumors. VII. Hepatic tumors. VIII. Malignant bone tumors. IX. Soft tissue and other extraosseous sarcomas. X. Germ cell tumors, trophoblastic tumors, and neoplasms of gonads. XI. Other malignant epithelial neoplasms and malignant melanomas. XII. Other and unspecified malignant neoplasms (including uncoded).

Children born January 1, 1990 were followed for 13 years and children born January 1, 2002 for one year. The counts of incident cases by histotype and year are listed in Table 1. Independent random walk priors were used to allow autoregressive temporal smoothing for each histotype. Temporal trends were readily identifiable and they varied considerably among histotypes. Two cancers with the greatest decrease in risk over the period of study were malignant bone tumors (e.g. osteosarcoma) and Hodgkin lymphoma. Two cancers with relatively steady risk over the study period were AML and "other leukemias." The temporal smoothing parameters used in the study are presented in Figure 4.

**Table 1:** Incidence by year and histotype

ICCC3 Group	1990	1991	1992	1993	1994	1995	1996	1997	1998	1999	2000	2001	2002
Ia.	107	90	98	90	92	80	101	82	75	60	49	33	11
Ib.	20	21	17	15	11	17	14	19	12	10	6	13	5
Ic, d, e,	7	12	12	11	4	8	3	4	6	9	7	10	7
IIa.	11	7	6	7	6	6	5	4	2	1	0	0	0
IIb, c, d, e.	22	18	29	21	11	19	16	14	14	15	7	5	3
IIIa.	7	9	15	8	6	10	6	10	8	7	5	6	3
IIIb.	39	35	32	27	39	31	31	33	21	13	11	7	3
IIIc.	15	15	25	16	20	20	15	12	8	9	8	9	4
IIId.	20	19	14	15	11	9	10	7	3	2	4	4	2
IIIe, f	30	20	15	18	12	15	6	10	7	9	8	7	4
IV.	26	27	31	22	29	31	32	42	32	33	30	20	19
V	20	13	16	18	5	13	12	12	16	15	17	15	7
VI.	23	16	31	28	11	29	22	20	28	27	13	14	6
VII.	3	8	9	5	7	7	7	9	8	9	3	5	2
VIII.	5	11	7	7	6	3	4	4	3	2	1	0	0
IX.	43	37	32	27	24	16	18	13	14	11	14	4	6
X.	8	11	15	12	10	12	7	14	7	6	8	11	6
XI.	18	9	6	4	3	5	4	4	1	0	4	1	1
XII.	4	7	7	1	2	4	0	2	2	2	4	2	0

International Classification of Childhood Cancer (ICCC3) Classification Key

Ia. Acute Lymphoid leukemias (ALL)

Ib. Acute myeloid leukemias (AML)

Ic, d, e, Other leukemias

IIa. Hodgkin lymphoma

IIb, c, d, e. Non-Hodgkin lymphoma

IIIa. Ependymoma and choroid plexus tumor

IIIb. Astrocytomas

IIIc. Intracranial and intraspinal embryonal tumors

IIId. Other gliomas

IIIe, f Other CNS tumors

IV. Neuroblastoma and other peripheral nervous cell tumors

V Retinoblastoma

VI. Renal tumors

VII. Hepatic tumors

VIII. Malignant bone tumors

IX. Soft tissue and other extraosseous sarcomas

X. Germ cell tumors, trophoblastic tumors, and neoplasms of gonads

XI. Other malignant epithelial neoplasms and malignant melanomas

XII. Other and unspecified malignant neoplasms (including uncoded)

For the combination of five geographical risk indicators and 19 cancer types, there were no SMRs whose 95% credible sets were above one. Hodgkin lymphoma appeared to be occurring with reduced risk in rapidly growing counties with > 90% of the posterior distribution less than one for SMR. There was support for an increased risk for hepatic tumors associated with high-release HAP locations and for germ cell tumors and "other" gliomas among high crop production locations. The median SMR and the 95% credible sets are listed in Table 2.

**Table 2:** Standard Morbidity Ratios for county characteristics of the mother's living location at the time of birth. Values are the median and 95% credible sets from the posterior distribution.

	CROPS	HAPS	METRO	URBAN	GROWTH
Acute Lymphoid leukemias (ALL)	1.01 (0.79, 1.28)	0.97 (0.76, 1.25)	1.04 (0.82, 1.36)	1.11 (0.82, 1.48)	0.97 (0.82, 1.16)
Acute myeloid leukemias (AML)	0.75 (0.41, 1.27)	0.81 (0.50, 1.29)	0.97 (0.61, 1.58)	1.01 (0.57, 1.84)	1.22 (0.87, 1.81)
Other leukemias	0.98 (0.50, 1.80)	0.57 (0.31, 1.02)	1.26 (0.66, 2.51)	1.60 (0.73, 3.71)	0.95 (0.58, 1.50)
Hodgkin lymphoma	1.00 (0.41, 2.36)	0.81 (0.35, 2.02)	1.03 (0.49, 2.40)	1.47 (0.52, 4.96)	0.49 (0.27, 0.96)
Non-Hodgkin lymphoma	1.02 (0.61, 1.70)	0.75 (0.48, 1.17)	1.10 (0.70, 1.77)	1.16 (0.68, 2.11)	0.88 (0.63, 1.26)
Ependymoma and choroid plexus tumor	0.56 (0.26, 1.12)	1.07 (0.59, 1.99)	0.90 (0.51, 1.60)	0.97 (0.46, 2.26)	0.86 (0.54, 1.39)
Astrocytomas	0.97 (0.67, 1.40)	0.80 (0.57, 1.15)	1.22 (0.85, 1.78)	1.07 (0.69, 1.71)	1.03 (0.79, 1.43)
Intracranial and intraspinal embryonal tumors	0.72 (0.43, 1.24)	1.12 (0.71, 1.77)	0.85 (0.55, 1.35)	1.42 (0.74, 2.80)	0.99 (0.69, 1.44)
Other gliomas	1.77 (0.98, 3.27)	1.41 (0.82, 2.54)	1.23 (0.69, 2.25)	1.08 (0.48, 2.72)	1.03 (0.64, 1.69)
Other CNS tumors	1.04 (0.57, 1.83)	0.94 (0.56, 1.55)	1.29 (0.78, 2.16)	0.82 (0.44, 1.65)	0.82 (0.57, 1.20)
Neuroblastoma and other peripheral nervous cell tumors	1.12 (0.78, 1.60)	1.15 (0.83, 1.59)	1.11 (0.81, 1.64)	0.74 (0.52, 1.09)	0.93 (0.74, 1.17)
Rtinoblastoma	0.86 (0.50, 1.48)	1.02 (0.60, 1.60)	1.22 (0.77, 1.99)	1.05 (0.58, 2.00)	0.89 (0.62, 1.32)
Renal tumors	0.95 (0.59, 1.51)	1.21 (0.80, 1.79)	1.04 (0.69, 1.61)	0.98 (0.58, 1.69)	0.91 (0.66, 1.28)
Hepatic tumors	0.80 (0.32, 1.91)	1.87 (0.95, 3.98)	0.99 (0.53, 1.90)	1.28 (0.46, 4.61)	1.16 (0.66, 2.18)
Malignant bone tumors	1.31 (0.51, 2.90)	1.15 (0.55, 2.55)	0.77 (0.36, 1.75)	0.67 (0.24, 2.08)	1.86 (0.89, 4.24)
Soft tissue and other extraosseous sarcomas	1.03 (0.64, 1.59)	0.86 (0.59, 1.28)	1.06 (0.70, 1.62)	1.42 (0.83, 2.57)	1.04 (0.74, 1.41)
Germ cell tumors, trophoblastic tumors, and neoplasms of gonads	1.54 (0.90, 2.75)	0.86 (0.50, 1.46)	1.40 (0.82, 2.64)	0.87 (0.46, 1.77)	1.03 (0.66, 1.63)
Other malignant epithelial neoplasms and melanomas	1.25 (0.57, 3.05)	0.89 (0.42, 1.97)	1.23 (0.60, 2.87)	0.98 (0.38, 3.01)	1.17 (0.66, 2.25)
Other and unspecified malignant neoplasms (including uncoded)	0.85 (0.25, 2.30)	0.68 (0.25, 1.75)	1.07 (0.47, 2.88)	1.17 (0.37, 4.83)	0.81 (0.37, 1.88)

**Figure 5:**
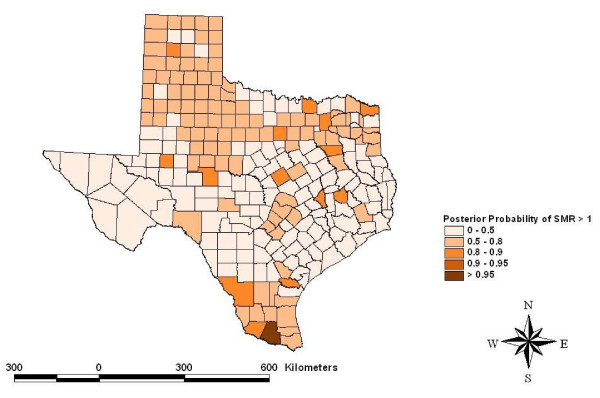
Spatial risks for "other" leukemias by county.

**Figure 6:**
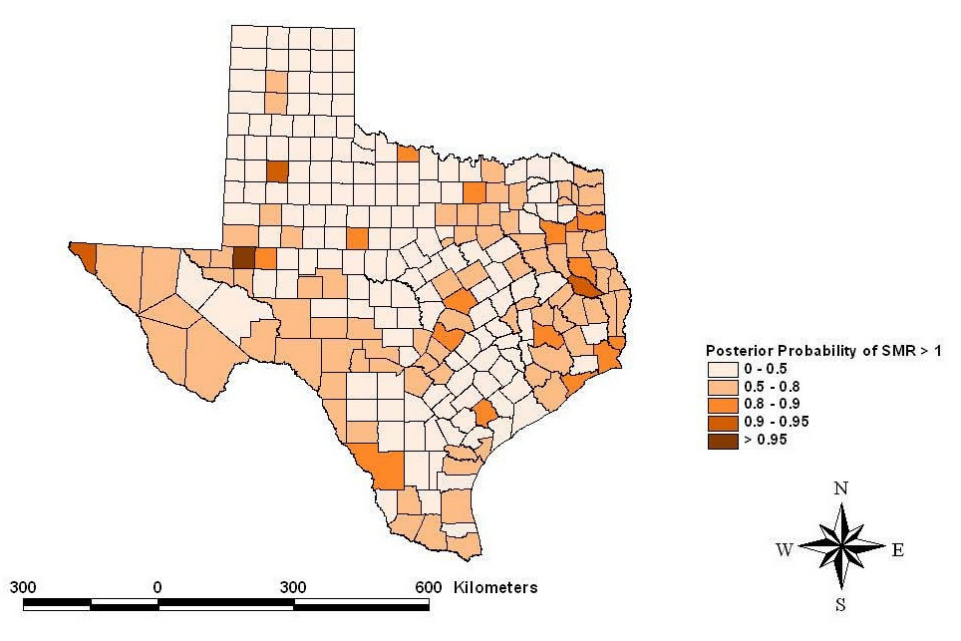
Spatial risks for CNS embryonal tumors by county.

**Figure 7:**
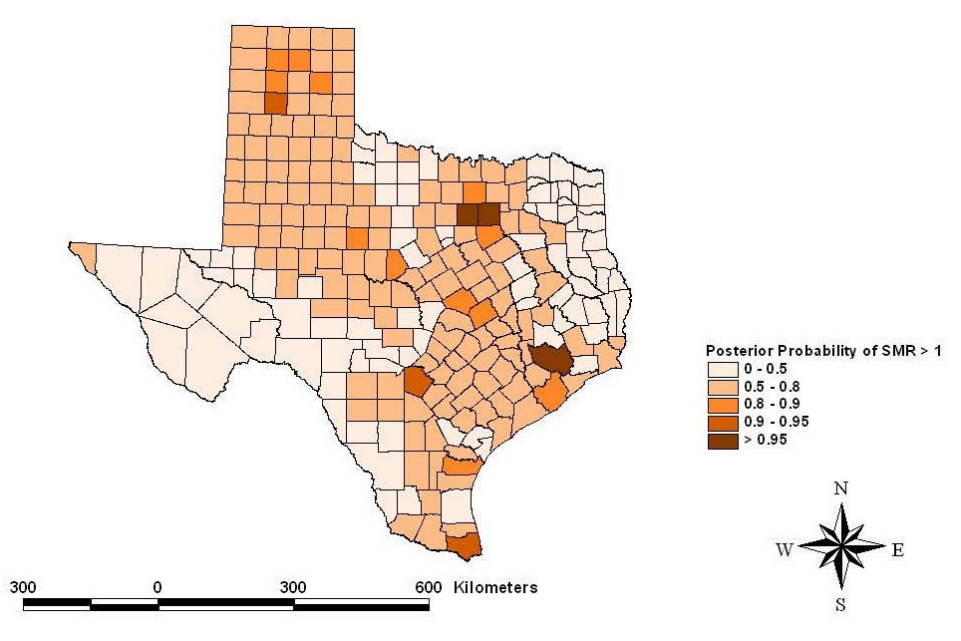
Spatial risks for CNS "other" gliomas by county.

**Figure 8:**
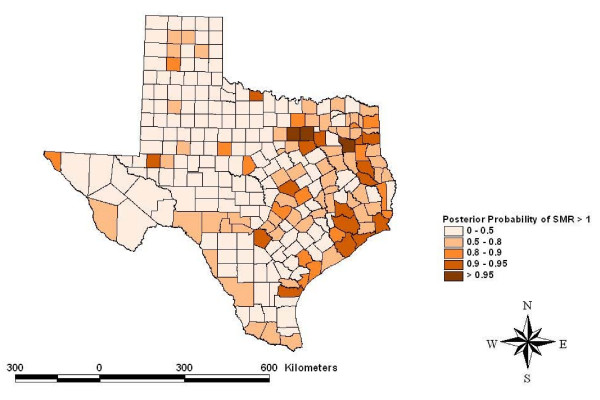
Spatial risks for hepatic tumors by county.

Risk maps identified counties for which the posterior likelihood of elevated SMR was greater than 95% for four cancers: other leukemias in Hidalgo County (Figure 5), CNS embryonal tumors in Ector County (Figure 6), CNS other gliomas in Parker, Tarrant and Harris Counties (Figure 7) and hepatic tumors in Parker, Tarrant and Smith Counties (Figure 8). Ten of 19 cancer histotypes had greater than 90% posterior probability of SMR greater than one for at least one county. The maps also showed spatial correlation among areas of elevated risk.

The correlations among histotypes and within county-years in the final model were generally near zero, ranging from -0.35 to 0.32.

## Discussion

The investigation reported here estimated personal risks for a child to develop cancer. This risk was defined by the mother's living location at the time of birth. Tumors with peaks in infancy were of special interest because they are more likely to have had causal exposures during the prenatal period. There are several childhood cancers known to have incidence peaks early in the infancy including neuroblastoma and other peripheral nervous cell tumors, retinoblastoma, renal tumors and hepatic tumors. Acute lymphocytic leukemia has a peak in infancy that is prominent among white children but less evident among black children. There are also histotypes with peaks in infancy and another peak later in childhood, including "other" leukemias and germ cell tumors, trophoblastic tumors and neoplasms of gonads [21]. Cancers with known incidence peaks in infancy showed temporal trends with relatively slow decrease in incidence for birth years 1990 to 2002. In contrast, the observed risk for cancers with incidence peaks in teenage years, Hodgkin lymphoma and malignant bone tumors [21] showed marked decline for the birth years 1990 to 2002. The temporal trends observed in the current study can be attributed to the latency period for the cancers and the variable period for follow-up. Although the primary exposure period of interest was the prenatal period for the current study, there is also interest in critical periods of exposure including earlier in gestation and the neonatal period. Also, it may be that many environmental exposures act not as tumor initiators, but as tumor promoters, so that exposures closer to diagnosis are also of interest. These were issues not addressed in the current study. Risk estimates were computed under a Bayesian paradigm maintaining sources of uncertainty in the risk estimates.

The county-level parameters were used as potential indicators of high-risk locations for further study and were selected from the conflicting evidence supporting their possible role as causes of childhood cancer. In general, it is not expected that the association between exposure and risk is linear for these geographic factors. The current analysis evaluated the risk of the extreme values for these potential indicators as observed in Texas. Cut-points for analysis were based on high values that allowed an adequate number of county-years (i.e., 15–20%) to be classified as "at risk." Even though Texas is considered an agricultural state there were only a low number of county-years with greater than 20% of the land area in intensive crop production. Studies in other locations may be able to evaluate a much higher cut-point. In contrast, the current study evaluated a very high cut-point of 100 tonnes of HAP. The population parameter cut-points for metropolitan and urban are used commonly by the U.S. census to classify counties. The identified factors could be related to many unknown potential causes thus the potential for confounding limits causal inference. It was the objective of this study to use county characteristics to focus further study. Once high-risk counties and their characteristics are identified, studies more specific to identifying environmental causes will become feasible.

The precision for geographic risk estimates has been especially problematic in the study of childhood cancer. It has been proposed that broader geographic regions could increase the precision of areal risk estimates for rare diseases [22]. However, aggregating of areal units will reduce the resolution of the GIS risk layer and will alter the relationship with a GIS exposure layer. Aggregation problems can result from the possibility of combining areal units that are actually very different in risk. The two problems created with using broader geographic regions, resolution and aggregation, are collectively known as the modifiable areal unit problem (MAUP). Combining spatial neighboring counts can be effective if the neighbors are very similar but the pooling would lead to non-differential risk classification if neighboring areal risks are dissimilar. Hierarchical approaches have been proposed to estimate the extent of correlation among neighboring locations and then adjust the risk estimates accordingly. The justification for Bayesian hierarchal modeling with vague priors is that the data likelihood will determine the extent of this pooling.

More specific causal studies should involve geographic risk modeling with a more precise geographic scale. The current study had available geocoordinates for individual births so it was theoretically possible to plot a continuous risk surface with a Bayesian geo-statistical approach [23] or more traditional approaches for cluster identification could have been used [24]. For the current study, the geographic factors were provided at the county level and, thus, dis-aggregation of the exposure to smaller geographic units, for example census tracts, could have led to an ecologic bias. However, TRI releases are available for point-source releases at specific geo-coordinates and detailed risk mapping in proximity to these sites is possible and should be the subject of further investigation. The current study identified locations for which this approach would most likely be rewarding.

In the posterior distribution, correlations among histotype pairs were small but ranged from moderately negative to moderately positive correlations. All non-zero correlations contribute to increased precision. The correlations were estimated fully conditionally on the geographic factors and were much smaller than in a previous study that did not identify attributes of specific locations [25]. As cancer risk modeling proceeds with geographic risk factors more precisely defined, the correlation among histotypes will eventually become attributable to specific geographic factors. The justification for a Bayesian approach and non-informative priors for spatial correlations among histotypes is that the approach can be used to increase comparability among studies. At present, the literature reveals a variety of ad hoc approaches to the grouping and parsing of childhood cancer histotypes. Previous epidemiologic studies have often used broad case definitions and frequently pooled data from multiple childhood cancer histotypes. The appropriateness of this pooling is largely unknown. Pooling cancer types with disparate causes will lead to a non-differential misclassification and usually increase the likelihood of a null finding. Failure to pool cancer types with common causes will lead to an unnecessary loss of precision. Specifying a flexible prior for the covariance matrix in a Bayesian approach can preserve this uncertainty or update the certainty based upon the data likelihood. Under Bayesian modeling, if two diseases are poorly correlated, the outcomes will remain relatively uncorrelated in the posterior distribution and the risk estimates will be the similar to estimates calculated independently for each histotype.

The current study supports further studies on germ cell tumors and other gliomas in areas with intensive cropping. Several studies have linked georeferenced disease counts and cropping patterns as a surrogate for pesticide exposure [7-10,26]. These studies varied widely on how cropping patterns were defined as exposure and how the childhood cancers, as a group of outcomes, were pooled or parsed. However, when risks of specific cancer types are evaluated subjectively among studies, the cumulative evidence supports the null finding. For the vast majority of childhood cancer types, the current study goes beyond a frequentist null conclusion by demonstrating SMR that were close to one with narrow 95% credible sets.

The current study supports the study of childhood hepatic cancer in areas of intense HAP release. The SMR for hepatic tumors was 1.87 (0.95, 3.98) for county-years with greater than 100 tonnes of HAP releases. Studies evaluating air pollution as a cause of childhood cancer have been inconsistent among a variety of cancer types [27]. The critical review showed several studies have evaluated multiple cancer types and groupings and found one or more histotypes at increased risk but other studies have found other histotypes at risk [27]. When individual cancer types are evaluated across studies, the cumulative evidence seems to support the null. Leukemia may be the exception, with some indication of increased risk among multiple studies of air pollution [28]. For cancer types other than hepatic cancer, the current study provides SMR estimates that center on no risk and have narrow confidence bounds, providing inductive support for the frequentist null results. Incriminating areal-source HAP concentrations in childhood cancer has been and will continue to be difficult. It has been reasoned that more definitive prospective studies should utilize biomarkers to study the risks of prenatal exposures [29-31]. Two recent studies illustrate the utility of biomarkers for studies defining the complex causal relationships between fetal exposures to air pollution and adverse outcomes [30,31]. Such an approach may be useful to study childhood hepatic cancer around major Texas industrial facilities.

The current study supports the investigation of Hodgkin lymphoma and malignant bone tumors in areas of rapid population growth. Hodgkin lymphoma is thought to be partly attributable to Epstein-Barr virus but also has genetic and environmental factors [32,33]. Low socioeconomic status increases risk for Hodgkin lymphoma [21] and it is possible that lowered risk observed in areas of rapid population growth in Texas could have been attributed to residents of higher socioeconomic status. Malignant bone tumors, including osteosarcoma as the most common of the class [34], had a high probability of increased risk in counties with rapidly growing population. Both Hodgkin lymphoma and osteosarcoma are considered to be more common in teenagers and the current study did not include any incident cases among teenagers. The risks seen for these two cancers in rapidly growing counties should receive more study.

Infectious causes and population mixing have been proposed as causes of childhood cancer [35]. The theory is that densely populated regions have high levels of herd immunity but populations with constant population mixing are at increased risk for individuals. The purpose of the current study was to evaluate the use of population characteristics for focusing further study. One study [17] found excess risk when population growth was greater than 10% in an eleven-year period, thus our risk definition of 1% per year. The population mixing theory does not parse the risk for those moving into a region from those already residing in the region and thus has only a population-based inference. For an individual deciding to move, the risks could be threefold. First, there could have been a geographic-based risk associated with the previous living location. Second, there could be a new geographic risk at the new living location. Third, there could be a risk of being a mover. The full evaluation of these risks would be complex and require hierarchical modeling if the objective included the estimation of risks interpretable at the individual mover level. The current study found median SMR for measures of population density and population growth to be very near one with narrow 95% credible sets for most childhood cancer types.

The spatial model identified counties with greater than 95% posterior likelihood of elevated SMR for specific childhood cancer histotypes. Hidalgo County had a high likelihood for increased SMR for atypical or "other" leukemias. Hidalgo County is a rapidly growing urban county on the Mexican border populated mainly by Hispanics. Ector County had a high likelihood for elevated SMR to CNS embryonal tumors. Ector County is an urban county populated relatively evenly by Hispanics and non-Hispanic whites. Three counties had a high posterior likelihood of elevated SMR to CNS "other" gliomas including Parker and Tarrant Counties collectively containing the Dallas/Fort Worth metropolitan area and Harris County which contains most the Houston metropolitan area. Both metropolitan areas are rapidly growing with considerable industrial development. Three counties had a high likelihood of elevated SMR for hepatic tumors including Parker and Tarrant Counties making up the Dallas/Fort Worth metropolitan area and Smith County. Smith County is an urban county but is often considered part of the Tyler metropolitan area. The risks estimated for these counties included the portion of the risk related to the factors that were evaluated and residual or random, unexplained geographic risks. Further study of these childhood cancer histotypes in these locations is indicated.

## Conclusion

The Bayesian implementation of the MCAR model provided a flexible approach to the spatial modeling of multiple childhood cancer histotypes. The approach parses the counts into specific counts of ICCC-3 classifications and flexible priors permit spatial smoothing and histotype correlations based on the data likelihood. Analysis of cancer risk by counties showed four cancer histotypes with greater than 95% likelihood of elevated SMR for further study. The identification of geographic factors supports more focused studies of germ cell tumors and "other" gliomas in areas of intense cropping, hepatic cancer near HAP release facilities and Hodgkin lymphoma and malignant bone tumors in counties with rapidly growing population.

## Competing interests

The authors declare that they have no competing interests.

## Authors' contributions

JT participated in the conception, design, analysis and drafted the manuscript. SC participated in the conception, data acquisition and helped draft the manuscript. LZ participated in the conception, design and analyses. All authors read and approved the final manuscripts.
